# BlindCall: ultra-fast base-calling of high-throughput sequencing data by blind deconvolution

**DOI:** 10.1093/bioinformatics/btu010

**Published:** 2014-01-09

**Authors:** Chengxi Ye, Chiaowen Hsiao, Héctor Corrada Bravo

**Affiliations:** ^1^Department of Computer Science, ^2^Center for Bioinformatics and Computational Biology and ^3^Applied Mathematics and Scientific Computing, University of Maryland, College Park, USA

## Abstract

**Motivation:** Base-calling of sequencing data produced by high-throughput sequencing platforms is a fundamental process in current bioinformatics analysis. However, existing third-party probabilistic or machine-learning methods that significantly improve the accuracy of base-calls on these platforms are impractical for production use due to their computational inefficiency.

**Results:** We directly formulate base-calling as a blind deconvolution problem and implemented BlindCall as an efficient solver to this inverse problem. BlindCall produced base-calls at accuracy comparable to state-of-the-art probabilistic methods while processing data at rates 10 times faster in most cases. The computational complexity of BlindCall scales linearly with read length making it better suited for new long-read sequencing technologies.

**Availability and Implementation:** BlindCall is implemented as a set of Matlab scripts available for download at http://cbcb.umd.edu/∼hcorrada/secgen.

**Contact:** hcorrada@umiacs.umd.edu

## 1 INTRODUCTION

Second-generation sequencing technology has revolutionized high-throughput genomics in life science and clinical research. The sheer scale of sequence generated by these instruments has allowed unprecedented views into a number of molecular phenomena, including population genetics, transcriptomics, epigenetics and translational profiling. Both the throughput and accuracy of second-generation sequencing instruments has increased at an accelerated pace in the last few years due to the use of high-resolution optics and biochemical methods that allow sequencing of billions of DNA fragments in parallel by generating fluorescence intensity signals that can be decoded into DNA sequences. However due to experimental and hardware limitations, these raw signals are inherently noisy (Aird *et al.*, 2011; Bravo and Irizarry, 2010; Dohm *et al.*, 2008; Erlich *et al.*, 2008). Base-calling is the essential step of converting these noisy fluorescent intensity signals into sequences used in downstream analysis. Providing accurate base-calls greatly reduces many difficulties in downstream bioinformatics analysis like genome assembly and variant calling (Alkan *et al.*, 2011; Bravo and Irizarry, 2010).

Sequencing-by-synthesis (Bentley *et al.*, 2008) generates millions of reads of short DNA sequences by measuring in parallel the fluorescence intensity of billions of PCR-amplified and labeled clusters of DNA from a sample of interest. The DNA fragments attach to a glass surface where it is then PCR-amplified *in situ* to create a cluster of DNA fragments with identical nucleotide composition. Sequence reads are generated from these DNA clusters in parallel and by cycles. A single nucleotide is sequenced from all DNA clusters in parallel by adding labeled nucleotides that incorporate to their complementary nucleotide. This synthesizes DNA fragments complementary to the fragments in each cluster as sequencing progresses. A set of four images is created measuring the fluorescence intensity along four channels to detect incorporation at each cycle. These images are then processed to produce fluorescence-intensity measurements from which sequences are then inferred by base-calling. In the default base-calling process for Illumina sequencers, called Bustard, the highest intensity in each quadruplet of intensity measurements determines the base at the corresponding position of the corresponding read. For current Illumina technologies, sequencers can produce up to 600 GB per run (Illumina, 2013).

**Fig. 1.**
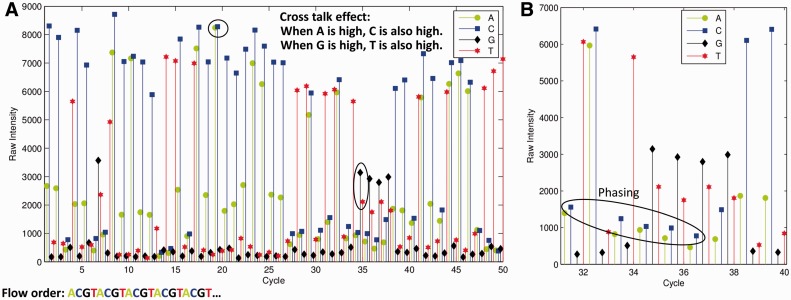
Signal properties in the base-calling problem. (A) Fluorescence intensity measurements from one cluster for 50 sequencing cycles. Cross-talk and signal decay effects are clearly observed in this data. Background intensity increases as sequencing progresses. (B) The phasing effect demonstrated on a subset of data from (A). High intensity in the C channel in cycle 32 affects background intensity in the C channel in neighboring cycles

The raw intensity signals generated by this process are known to be subject to several biases (Aird *et al.*, 2011; Bravo and Irizarry, 2010; Dohm *et al.*, 2008; Erlich *et al.*, 2008) (Fig. 1A and B). (i) Cross talk: there are significant correlations between different nucleotide channels; (ii) phasing/pre-phasing: the signal in one cycle can spread to the cycles ahead and the cycles after it; (iii) signal decay: where signal intensities become lower in later sequencing cycles; (iv) background noise: the signal to noise ratio becomes lower in later sequencing cycles. A significant challenge in base-calling is accounting for these biases.

Existing base-calling methods can be classified into two major groups: (i) unsupervised model-based methods that capture the sequencing-by-synthesis process in a statistical model of fluorescence intensity from which base-call probabilities can be extracted directly (Bravo and Irizarry, 2010; Kao and Song, 2011; Kao *et al.*, 2009; Massingham and Goldman, 2012) and (ii) supervised methods that train a statistical model on a set of base-calls whereby fluorescence intensity measurements are classified into base-calls (Erlich *et al.*, 2008; Kircher *et al.*, 2009). The former methods have been shown to significantly improve the accuracy of Bustard base-calls. These model-based methods aim to capture the sequencing process described above in a statistical model from which base-call probabilities are usually obtained. While these probabilistic or machine-learning methods improve the accuracy of base-calls, they are impractical for use due to their computational inefficiency, which usually scales quadratically with read length since most of them resort to dynamic programming for model fitting (Kao and Song, 2011; Kao *et al.*, 2009; Massingham and Goldman, 2012).

In this article, we show that the base-calling problem can be formulated as an optimization problem called blind deconvolution. Based on this observation, we developed BlindCall as a method that treats base-calling as a blind deconvolution problem (Levin *et al.*, 2011; Xu *et al.*, 2013). We model intensity signals (*B*) output by the sequencer as the convolution of a latent sparse signal of interest *X* and a convolution kernel *k* modeling cross-talk and phasing biases, plus background noise *N*:

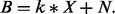



The blind deconvolution problem is to recover the latent signal *X* given only the observed *B*. This reduces the base-calling problem into solving an inverse problem that admits computationally efficient solutions. The blind deconvolution problem has been a research hotspot in recent years (Levin *et al.*, 2011; Xu *et al.*, 2013) and we adapt methods for its solution to the base-calling problem (Wang and Yin, 2010).

BlindCall was able to provide base-calls at comparable accuracy to state-of-the-art probabilistic methods while processing data at rates ten times or faster in most cases. It scales linearly with read length and is thus better suited for new long-read sequencing technologies. Direct blind deconvolution modeling and the ultra-efficient processing based on optimization methods presented here are essential for bioinformatics analysis workflows to cope with increased throughput and read lengths in new sequencing technologies.

## 2 METHODS

**Fig. 2.**
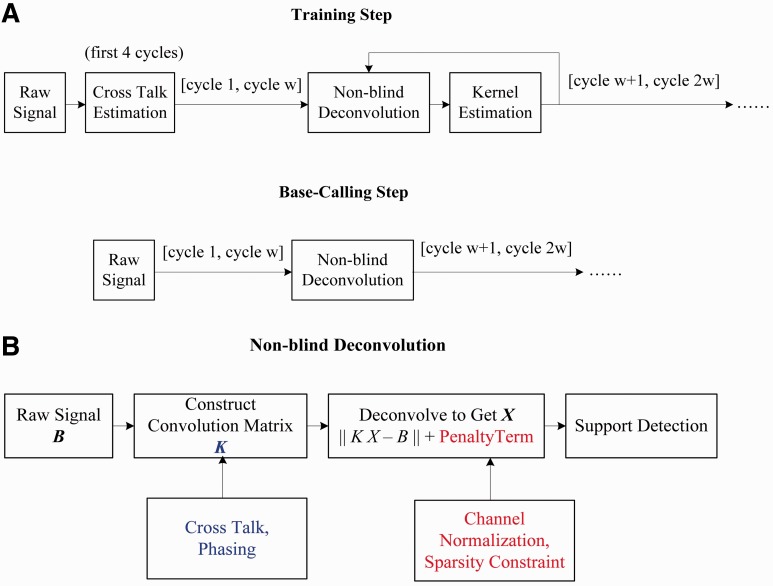
The BlindCall architecture. BlindCall consists of two modules: (A) the training module uses blind deconvolution and (B) to simultaneously estimate model parameters and produce a deconvolved signal from which base-calling is done. The calling module uses the parameters estimated in the training module to produce a deconvolved output signal

BlindCall follows the following architecture (Fig. 2A): a training module uses blind deconvolution (Fig. 2B) on a randomly sampled subset (e.g. 1000 reads) of the intensity data to iteratively estimate the convolution kernel *k* and produce a deconvolved signal from which base-calling is performed. The base-calling module then uses the convolution kernel estimated in the training module to produce a deconvolved output signal for the entire dataset and call bases.

### 2.1 Blind deconvolution

We solve the blind deconvolution problem using an iterative procedure: (i) fixing *k* and estimating latent signal *X* using a specific non-blind deconvolution method based on iterative support detection (ISD) (described below) and then (ii) fixing *X* to estimate convolution kernel *k* to correct for cross-talk and phasing effects. We divide the signal into non-overlapping windows: in each 20-cycle window we assume an invariant convolution kernel. The discrete convolution can be written as matrix multiplication *B* = *KX*, where *K* is a convolution matrix constructed from the kernel *k*. A normalization procedure is used in each iteration to account for intensity biases across channels.

### 2.2 Channel intensity normalization

Intensity data for Illumina sequencing show certain biases, specifically (i) signal strength variation across channels, (ii) signal strength variation across clusters and (iii) signal decay over sequencing cycles. For accurate base-calling, these biases must be addressed through normalization. Traditionally, read normalization is applied to tackle the second and third problems first, in order to address the first problem. In our method, we circumvent the read normalization problem by analyzing the relative intensity ratio of successive calls across sequence reads.

After an initial deconvolution in which cross-talk is corrected, we normalize each channel by scaling the intensities across reads by the same quantile (95%) in the respective channels and select the strongest channel after normalization as candidate base-calls. We then select successive candidate calls that are of different bases and construct a set of linear equations of the form 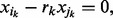 where 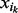 and 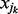 are the relative intensity of channels in the *k*-th relation and 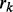 is the observed intensity ratio for the *k-*th relation. The set of linear equations is then 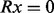, where *R* is a 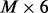 matrix, with *M* being the total number of base-calls pairs within consideration. To estimate *x*, we solve a least-squares problem under the constraint that 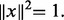 The solution is obtained by solving an eigenvalue problem since it can be formulated into the Rayleigh quotient 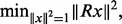 and its solution must satisfy the eigenvalue equation 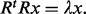 Since the number of base-calls across channels varies, the solution of this optimization problem favors channels that are called frequently. We normalize the problem using the number of base-calls and solve the generalized eigenvalue problem 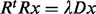 where 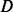 is a diagonal matrix that records the number of base-calls in each channel. This formulation can be interpreted as finding the stable state of a normalized non-linear diffusion, and is used in normalized cut (Shi and Malik, 2000), Laplacian Eigenmaps (Belkin and Niyogi, 2001), and PageRank (Page *et al.*, 1999). The estimated vector *x* is the relative intensity of each channel and we use it to normalize each channel in subsequent steps.

### 2.3 Sparse signal reconstruction through ISD

To perform base-calling we need to reconstruct latent sparse signal *X*, corresponding only to nucleotide incorporation measurements given a convolution kernel *k*. A straightforward 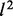 optimization problem to estimate latent signal *X* minimizes 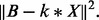 We know the latent signal is sparser than the observed signal, so we add this property as a constraint to the least squares problem and use an iterative procedure to solve the problem under the sparsity constraint. This idea is termed ISD in the mathematical community (Wang and Yin, 2010), and can also be applied to deconvolution problems stemming from image deblurring applications. In our case, the support (non-zero entries) detected for latent signal *X* corresponds exactly to base-calls. Assuming 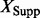 is the signal taking non-zeros only in the support set obtained using our support detection algorithm, we want to find an *X* that minimizes 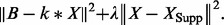 This optimization outputs a corrected signal subject to the support set constraint. The support detection procedure is critical to the output accuracy—if the support set is correct, we are close to our solution. At the beginning, we have no knowledge of the support set, since that directly tells us the answer. To tackle this, we use an increasing series 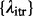 that puts increasing weight on the second constraint. This weight is low at first since the support set is not accurate. As we gradually refine the estimates we increase this weight. In our implementation, support detection is conducted by incorporating the channel-normalization method discussed in the previous section and picking the strongest normalized channel.

We provide further mathematical justification as to why this iterative procedure recovers the clear intensity signals of incorporation events. For reference to the applications in image deblurring we refer to the convoluted signal *B* as the blurred signal, and to the latent signal *X*, the clear signal.

Observation 1: Assume the clear signal is a non-negative signal with spikes, the convolution (blur) kernel is non-negative and 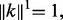 then the convoluted (blurred) signal is denser than the latent (clear) signal.

This observation holds for all blurs since the blur spreads the spikes thus creates more non-zero intensities, so the support set becomes larger with the blurred signal. This observation hints us to design an optimization that favors sparse solutions:

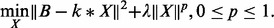



The second term is a sparse-inducing penalty. This sparse regularization problem is well known in wavelet analysis (Mallat, 2009). We also have the following observation.

Observation 2: By comparing the 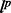 norm 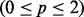 of the clear/blurred signal, we discover that the sparse norm penalty favors the clear signal.

As special cases:

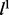
norm measures the total variation of the signal, thus the blurred signal and clear signal have the same 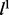 norm.
- The 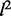 norm of the blur signal is smaller than that of the clear signal.
- The support set for the blurred signal is larger than the clear signal, therefore it has larger 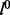 cost.

The above observations suggest that we use a sparse norm to penalize the blur signal and make it resemble the clear signal. Thus, we analyze the deconvolution model with an 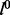 penalty:

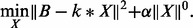



By introducing an auxiliary variable and using an exterior penalty technique, the above minimization problem is equivalent to solving the following optimization problem:

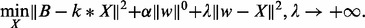



One strategy to solve the above optimization is the alternating minimization technique (Wang *et al.*, 2008) and cast the problem into two sub-problems: (i) fixing *X* and analyzing the terms containing *w*, we have the *w* sub-problem:

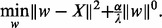



The solution can be found by entry-wise comparison (Mallat, 2009; Xu *et al.*, 2013) and the result is the so-called hard thresholding:

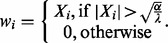



Then (ii) fix *w*, and analyze the terms containing *X*, we have

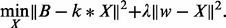



This optimization problem has the same form with our deconvolution model when 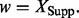 In our ISD method, 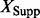 is obtained by adaptive hard thresholding, where *α* is set adaptively to select strictly one non-zero element into the support set by selecting the channel with maximum intensity. Thus, our ISD method solves an optimization problem with an 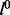 penalty favoring sparse signals corresponding to nucleotide incorporation.

### 2.4 Convolution kernel estimation

Given latent signal *X* we use a least-squares method to estimate the convolution kernel *k* modeling cross-talk and phasing effects by solving:

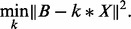



We estimate convolution kernel *k* in two distinct steps: we use data from the first four cycles and only model cross-talk in the convolution kernel and use the blind-deconvolution iterative procedure to estimate cross-talk effects. We then fix the components of the convolution kernel corresponding to cross-talk effects for the remaining windows and estimate the components of the convolution kernel corresponding to phasing effects only. We assume the phasing effect is the same across channels.

### 2.5 Deriving quality scores from deconvolved signal

We measure the quality of a base-call by the ratio of the intensity of the strongest channel and the sum of the two strongest channels after the deconvolution procedure. This number ranges between 0.5 and 1.0 and is used as the raw quality score. This scheme is similar to the one in Illumina’s Bustard basecaller. Like most existing base-callers, we calibrate these raw quality scores by aligning reads to the reference genome and mapping raw quality scores to the alignment error rate.

### 2.6 Validation methods

The following datasets were used to test the accuracy and computational efficiency of BlindCall and state-of-the-art probabilistic methods:

*Illumina HiSeq 2000 phiX174*: 1 926 928 single-end reads of 101 cycles from a single tile. Data was sequenced at the University of Maryland, College Park and is available for download at http://cbcb.umd.edu/∼hcorrada/secgen.

*Ibis Test*: 200K single-end reads of phiX174 >51 sequencing cycles.

*Bordetella pertussis*: 100 tiles of 76-cycle single-end reads from the coccobacillus *B.pertussis*, using the complete genome of the Tohoma I strain as a reference.

*AYB phiX174*: released with AYB and contains human sequence with a PhiX174 spike-in.

The last three datasets were downloaded from the AYB authors’ website (http://www.ebi.ac.uk/goldman-srv/AYB/#data).

To calculate accuracy we align the reads based on the phiX174 reference using Bowtie2 (Langmead and Salzberg, 2012) with –end-to-end and –sensitive settings. Reported error rates are based on reads with no more than five substitution errors, following the methodology in Massingham and Goldman (2012). We used SparseAssembler (Ye *et al.*, 2012) to obtain assemblies from base-calls obtained by each method. To derive assembly statistics, we sub-sampled 100 datasets from the complete set of reads at 5×, 10× and 20× coverage, and perform assemblies on each of these. We report N50 and maximum contig length for each resulting assembly.

Version 1.9.4 of the Off-line basecaller was downloaded from Illumina to run Bustard. Version 2 of AYB was downloaded from http://www.ebi.ac.uk/goldman-srv/AYB. We ran AYB for 5 iterations as per its default setting.

## 3 RESULTS

BlindCall is implemented as a set of Matlab scripts available at http://cbcb.umd.edu/∼hcorrada/secgen. As an example of its computational efficiency, running BlindCall on a single-core Matlab instance on an Intel i7 3610QM laptop with 2.3–3.3 GHz processor and 8 GB of memory, we found that it was able to process 1 million bases/s, or >85 billion bases/CPU day. We note that a significant portion of its running time (50%) is spent on disk IO to read intensity data and write the fasta/fastq outputs. To the best of our knowledge, BlindCall is one of the fastest base-callers available at this time, even though it is implemented in a scripting language. A port of this algorithm into a lower-level language (C/C++) will give further improvements on speed over the current Matlab version.

**Fig. 3.**
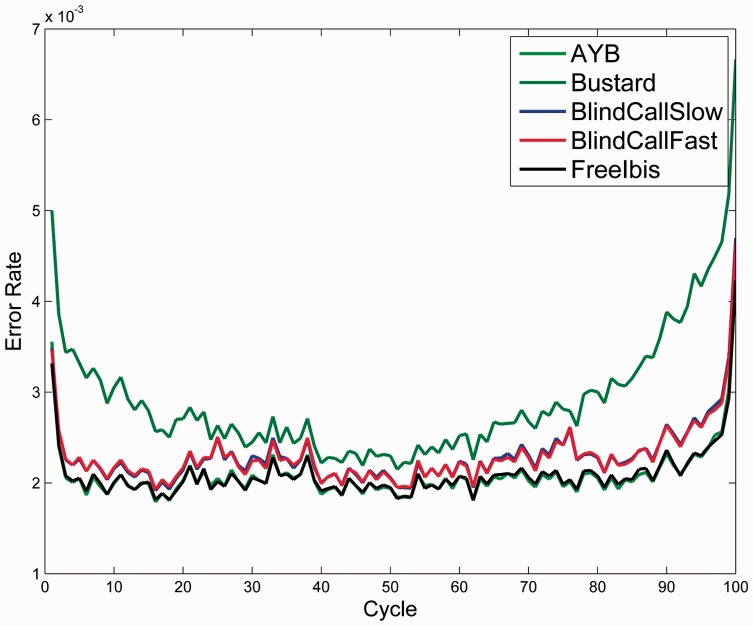
Third-party base callers improve Bustard per-cycle error rate. We plot error rate of each base-caller per sequencing cycle on the PhiX174 test data. All three base callers significantly improve accuracy over Bustard, especially in later cycles. BlindCall is able to achieve comparable accuracy while processing data at a much faster rate

We compared the running time of BlindCall to the state-of-the art probabilistic base-caller AYB (Massingham and Goldman, 2012) and the state-of-the-art supervised learning method freeIbis (Renaud *et al.*, 2013) on a dataset of 1.9 million reads from a PhiX174 run on an Illumina HiSeq 2000 (Table 1). We found that BlindCall was able to process this dataset ∼20 times faster than AYB and 10 times faster than freeIbis while retaining similar accuracy. A plot of per cycle error rate of these base-callers (Fig. 3) shows that all methods produce significant improvements over Bustard, especially in later sequencing cycles. We observed a similar pattern when testing other datasets (Table 2).

**Table 1.** Base callers accuracy and runtime comparison

	Bustard		AYB		BlindCall slow		BlindCall fast		freeIbis	
Perfect reads	1 446 079		1 532 000		1 509 451		1 508 779		1 530 099	
Error rate (%)	0.29		0.21		0.23		0.23		0.21	
Time (minimum)	17		217		8/12		4/8		9/126	
Assembly results	N50	Maximum	N50	Maximum	N50	Maximum	N50	Maximum	N50	Maximum
5×	610	1122	628	1155	629	1164	623	1167	649	1184
10×	3 375	3469	3198	3322	3382	3487	3389	3485	3306	3418
20×	4466	4478	4627	4637	4511	4523	4470	4483	4333	4357

AYB, accuracy and run times for Bustard. freeIbis and BlindCall for a dataset of 1.9 million reads from a HiSeq 2000 run of PhiX174. BlindCall Fast corresponds to non-iterative version of the blind-deconvolution method. Running times for BlindCall are reported as (processing time/total time), where the total time includes reading intensity data from disk and writing base-calls to disk. For freeIbis, we report the time as (predicting time with single thread/ training time with 10 threads). BlindCall was able to produce base-calls of comparable accuracy to AYB and freeIbis at significantly faster computational time (8 min/12 min versus 217 min and 126 min, respectively). It is also faster than Bustard (8 min/12 min versus 17 min). AYB, freeIbis and BlindCall all improve on Bustard base calls. We also compared assemblies of the PhiX174 genome using reads generated by Bustard, BlindCall, freeIbis and AYB. The reported N50s and Max contig lengths are averages >100 random samples with the corresponding coverage (5×, 10× or 20×). While BlindCall is able to process data at a significantly lower computational cost, the assemblies obtained using BlindCall are of comparable quality to those obtained using AYB or freeIbis.

**Table 2.** Accuracy comparison

		Ibis Test		*B.pertussis*		PhiX174 (AYB)	
		Perfect reads	Error rate (%)	Perfect reads	Error rate (%)	Perfect reads	Error rate (%)
Bustard		99 834	1.45	1 557 963	2.01	24 478	0.49
AYB		133 537	0.73	2 304 005	1.26	26 878	0.38
BlindCall slow		110 951	1.12	1 902 621	1.61	25 144	0.45
BlindCall fast		105 312	1.26	1 856 286	1.66	24 740	0.47
Time	Slow	0.08/0.3/1		0.11/6/10		0.15/14/22	
	Fast	0.08/0.1/1		0.11/3/8		0.15/7/16	

Accuracy for Bustard, AYB and BlindCall on various datasets. BlindCall was able to produce comparable accuracy to state-of-the-art base callers at significantly faster computational time. All methods improve on Bustard base calls. Run times for BlindCall are reported as (training time/processing time/total time in minutes) where the total time includes reading intensity data from disk and writing base-calls to disk.

**Fig. 4.**
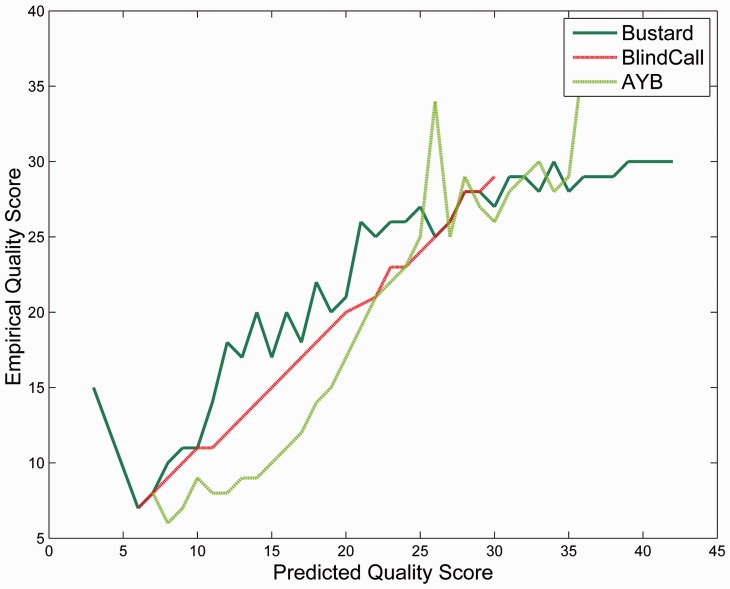
BlindCall produces accurate calibrated quality scores. We plot observed error rates (on the PHRED scale) for Bustard, AYB and BlindCall as predicted by quality scores and observed high correlation for all base callers

We also obtained better assemblies, especially at low coverage, using BlindCall, AYB and freeIbis relative to Bustard base-calls (Table 1). We also found that the calibrated quality values obtained from BlindCall are very accurate (Fig. 4).

**Fig. 5. btu010-F5:**
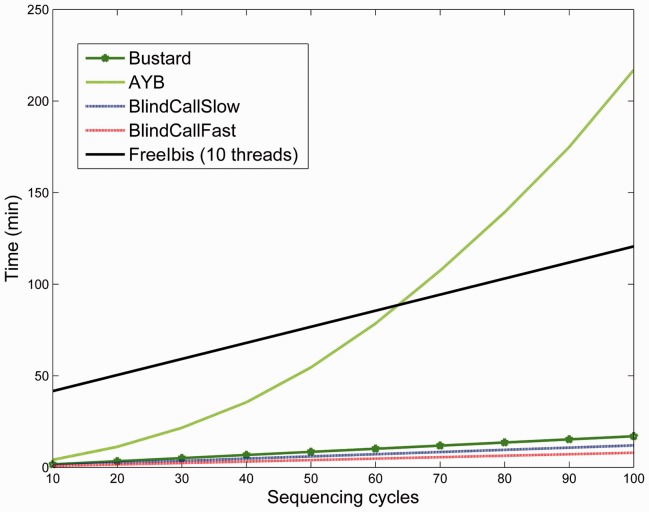
Base-calling by blind deconvolution is scalable to long read lengths. We compare the computational time of BlindCall with a state-of-the-art probabilistic base caller AYB, the state-of-the-art supervised learning method freeIbis and Illumina’s Bustard on the PhiX174 dataset reported in Table 1 as a function of the number of sequencing cycles. Since most model-based base callers resort to a dynamic programming solution, running time is quadratic with respect to the read length. In contrast, BlindCall scales linearly with read length. Base callers based on the blind deconvolution framework will be able to scale as sequencers produce longer reads. freeIbis also scales linearly but is much slower than BlindCall

We next compared each base-calling method’s ability to scale to longer read lengths by calculating running time as a function of read length for the same dataset (Fig. 5). Like most probabilistic model-based base callers, AYB resorts to a dynamic programming strategy with quadratic running time complexity with respect to the read length. In contrast, BlindCall scales linearly with read length. freeIbis uses supervised learning approach, and while it also scales linearly with read length, its training time is much slower than BlindCall (even using 10 threads for freeIbis, compared to a single thread for BlindCall). Base-callers based on the blind deconvolution framework will be able to scale as sequencers produce longer reads.

## 4 CONCLUSION

BlindCall is a simple and ultra-fast non-probabilistic base-calling method for Illumina high-throughput sequencing data based on blind deconvolution. We have shown that it provides comparable accuracy to probabilistic base-calling methods while producing base-calls at rates more than ten times faster.

Almost all probabilistic methods solve the base-calling problem in a ‘forward’ way, i.e. by setting a set of basis functions and searching for an optimal path, which often leads to dynamic programming solutions. Fitting these statistical methods is computationally expensive, and will not scale as the increase in sequencing throughput continues. Also, a stationarity assumption must be made in order to estimate parameters in these probabilistic methods through a Markov process. In contrast, BlindCall models base-calling as an ‘inverse’ problem of blind deconvolution, which requires no probabilistic assumptions of the sequencing process.

As steady progress has been made to improve the accuracy of probabilistic methods, we expect that similar progress will be made on non-probabilistic methods based on the blind deconvolution methods described in this article. Furthermore, these methods will be better suited to cope with increased throughput and read lengths of new sequencing technologies.[Fig btu010-F5]
